# Speech-Language Pathology and Audiology in South Africa: Clinical Training and Service in the Era of COVID-19

**DOI:** 10.5195/ijt.2021.6376

**Published:** 2021-06-22

**Authors:** Katijah Khoza-Shangase, Nomfundo Moroe, Joanne Neille

**Affiliations:** 1 Department of Speech Pathology and Audiology, School of Human and Community Development, University of the Witwatersrand, Johannesburg, South Africa

**Keywords:** COVID-19, South Africa, Telepractice, Teletraining

## Abstract

**Introduction and purpose:**

The novel coronavirus (COVID-19) presented new and unanticipated challenges to the provision of clinical services, from student training to the care of patients with speech-language and hearing (SLH) disorders. Prompt changes in information and communication technologies (ICT), were required to ensure that clinical training continued to meet the Health Professions Council of South Africa's regulations and patients received effective clinical care. The purpose of this study was to investigate online clinical training and supervision to inform current and future training and clinical care provision in SLH professions.

**Methodology:**

A scoping review was conducted using the Arksey and O’Malley (2005) framework. The electronic bibliographic databases Science Direct, PubMed, Scopus, MEDLINE, and ProQuest were searched to identify publications about online clinical training and supervision and their impact on clinical service during COVID-19. Selection and analysis were performed by three independent reviewers using pretested forms.

**Results and Conclusions:**

The findings revealed important benefits of teletraining and telepractice with potential application to South African clinical training and service provision. Five themes emerged: (1) practice produces favorable outcomes, (2) appreciation for hybrid models of training and service delivery, (3) cost effectiveness is a “big win” (4) internationalization of remote clinical training and service provision, and (5) comparable modality outcomes. These findings may have significant implications for teletraining and telepractice in low-and-middle income countries (LMICs) in the COVID-19 era and beyond, wherein demand versus capacity challenges (e.g., in human resources) persist. Current findings highlight the need for SLH training programmes to foster a hybrid clinical training model. Few studies were conducted in LMICs, indicating a gap in such research.

At the inception of COVID-19, the speech-language and hearing professions’ (SLH) clinical training and clinical service provision in South African training institutions were largely traditional and conservative in nature. Most clinical encounters occurred via in-person contacts with limited use of teletraining and telepractice, despite the well documented shortages of providers in South Africa. Published evidence within the South African context is increasingly agitating for change and re-imagination of how training as well as service provision is provided (Khoza-Shangase & Mophosho, 2018, in press; Koens et al., 2020; Pillay & Kathard, 2018). Within SLH, Abrahams et al. (2019) gleaned that increasingly literature focusing on clinical service provision is reflecting: (1) the need for transformation of the professions, depicted by the recognition of the importance of acknowledgement of the impact that linguistic and cultural diversity has on outcomes, coupled with development of culturally and linguistically appropriate assessment and intervention tools (Barratt et al., 2012; HPCSA, 2019; Khoza-Shangase & Mophosho, 2018; Mdlalo et al., 2019); (2) the need for SLH practitioners to consider their own positionality in relation to the racial, linguistic and cultural diverse populations served (HPCSA, 2019; Khoza-Shangase & Mophosho, 2018); and (3) the requisite to think creatively when considering the needs of the South African context (Khoza-Shangase & Moroe, 2020; Maluleke et al., 2021; Moonsamy et al., 2017; Naude & Bornman, 2021).

Khoza-Shangase and Mophosho (2018) describe the South African context under which SLH practitioners provide clinical services and highlight a number of challenges. These challenges include (1) well-documented lack of appropriate skills, with the handful of training institutions graduating, on average, less than 300 SLH practitioners a year; (2) unfavourable professional-to-patient ratios in terms of demand versus capacity with obvious incongruence between majority English and/or Afrikaans speaking SLH practitioners and 80% non-English speaking population; (3) lack of universal healthcare coverage due to infrastructural constraints where access to health care services is severely compromised by limited well-functioning public health facilities; (4) general lack of resources for the size of the population requiring health services – including SLH services; (5) lack of proper SLH epidemiological data that allows for risk versus benefit assessments for all SLH services; and (6) documented evidence of limited implementation of best practice clinical guidelines and translation of knowledge and policies into practice for various reasons including linguistic and cultural diversity quandaries as well as lack of political will.

Most recently, Abrahams et al. (2019) added inequality and professionalization as additional challenges under which South African SLH practitioners must function. These authors argued that SLH service delivery in South Africa continues to be inequitable over two decades post-apartheid, with clinical practice in its traditional form still adopting largely an individual focus – which serves as one key barrier to SLH services access. These authors, supported by Mophosho et al. (2019), Overett and Kathard (2006), Pascoe et al. (2010), and, Pillay and Kathard (2015), conclude that speech-language pathology and audiology services are accessed by mainly middle class, generally white populations who speak a dominant language such as English. Consequently, the underserved population is therefore largely poor, Black, African language speaking, and lives in rural and semi-rural areas (Khoza-Shangase, 2021). Abrahams et al. (2019) assert that the current state of unattainable, unaffordable, and inaccessible clinical services within the South African context, particularly for the majority of the population, perpetuates systemic marginalisation of the majority of South Africans. Hence the strong argument for considerations of different service delivery models to achieve universal SLH services coverage (Khoza-Shangase, 2021), taking advantage of the National Health Insurance (NHI) Bill of 2019, whose goal is that of the World Health Organization's universal health care coverage (Ghebreyesus, 2017; WHO, 2017).

The well documented challenges regarding capacity versus demand as far as SLH practitioners and the South African population with communication and swallowing disorders, begs for a paradigm shift in how SLH services are provided within this context, both within the clinical training and clinical service provision platforms. Khoza-Shangase and Sebothoma (in press) recommend that one of the key shifts in thinking involves use of different service delivery models, such as telehealth/telepractice, as well as task shifting in service provision. These authors argue that sensible application of current and emerging technologies to deliver SLH services can assist in providing specialized expertise not otherwise available, enhance clinicians’ productivity, and improve access to quality services in a cost-effective manner, while utilising paraprofessionals in task shifting. Within clinical training, the authors of this manuscript support these benefits with additional benefits of access to clinical supervision from supervisors from across the world, facilitating clinical training in training platforms far removed from the university (widening access), and possibly increasing training as well as supervision to the rest of Africa where SLH training is not available. Telepractice and teletraining are options requiring serious deliberation within SLH professions in low- and-middle-income countries (LMICs), such as South Africa, not only to address the demand versus capacity challenge; but also, as a response to the direct contact restrictions brought by COVID-19. Benson (2020), for example, has reported that audiological assessment during COVID-19 should include video otoscopy, tympanometry and pure tone audiometry; which can all be conducted through real-time synchronous and asynchronous tele-audiology (Coco, 2020; Khoza-Shangase & Moroe, 2020). Ohannessian et al. (2020) highlight that COVID-19 has also demonstrated the use of global health teleconsultations and tele-expertise as part of telepractice. Therefore, careful consideration of telepractice/teletraining as a platform to deliver speech-language pathology and audiology services in the advent of COVID-19 (and beyond), with its social distancing requirements, makes this imperative and an urgent need for LMIC contexts.

On 31 December 2019, the World Health Organization (WHO) reported a cluster of pneumonia cases in Wuhan City, China. ‘Severe Acute Respiratory Syndrome Coronavirus 2’ (SARS-CoV-2) was confirmed as the causative agent of what is now known as ‘Coronavirus Disease 2019’ (COVID-19). Since then, the virus has spread to 220 countries globally, 47 of which are in Africa including South Africa (WHO, 2020). Because COVID-19 is reported to have spread globally due to a lack of prior immunity combined with its relatively high infectiousness, a significant part of preventing its spread has been around implementing public health measures to reduce transmission (Karmelin & Kasson, 2020; Li et al., 2020; Wölfel et al., 2020; Zhou et al., 2020). Karmelin and Kasson (2020) describe how, in advance of effective vaccines and therapies for COVID-19, countries have adopted different public health measures to reduce transmission. These have been classified as “*suppressive* approaches, which aim to arrest transmission, and *mitigation* approaches, which aim to slow spread and shield vulnerable populations without truncating transmission” (p.1). Social distancing and ventilation have been emphasized broadly to control the ongoing pandemic COVID-19 with warnings having been issued that close contact should be avoided on account of virus transmission via droplet and airborne routes by respiratory activities (Sun & Zhai, 2020). This method of reducing transmission, particularly with the type of patient population seen by SLH professions, a majority of which fall under the vulnerable groups (Kuy et al., 2020), called for changes in clinical training as well as clinical service provision to that of teletraining and telepractice.

The use of teletraining and telepractice has increasingly been documented internationally. This has included their co-use in teaching both students and patients in assessment and management of chronic diseases within communities in primary healthcare (Kolltveit et al., 2016, Steventon et al., 2016), in assessment of both students and patients (Cassel & Edd 2016; Giudice et al., 2015), in clinical supervision (Batthish et al. 2013; Cameron et al., 2015; Harris, 2020) – through the use of various measures including telephones (Bunker et al., 2017; Thomas et al., 2021), online access (Muflih et al., 2020); and videoconferencing (Cameron et al., 2015; Howells et al., 2019), to name a few. The COVID-19 pandemic has raised the need for thorough deliberations around this method of service and training delivery (Chandrasinghe et al., 2020; Grewal et al., 2020; Muflih et al., 2020), hence the current study.

## METHODOLOGY

Adhering to the methodology advocated by Levac et al. (2010), the research team was comprised of three researchers working in academia in the fields of speech-language pathology and audiology. They agreed upon the research question, the search terms, keywords, and phrases to be searched, and the searchable databases. The researchers adopted the Arksey and O’Malley's (2005) five phased framework: (1) identifying the research question, (2) identifying relevant publications, (3) study selection, (4) charting the data and (5) collating, summarizing, and reporting the results.

### RESEARCH QUESTION

This review explored the question: ‘Is telepractice useful for clinical training and clinical service delivery?’ This question was guided by the increasing need for the use of ICT globally in all sectors of societies – particularly within healthcare delivery. COVID-19 accelerated the use of telehealth as a strategy to lessen the pandemic's spread. The researchers reviewed the available evidence to identify barriers for teletraining and telepractice within the South African SLH professions. Furthermore, influenced by Daudt et al. (2013) on the value of scoping reviews, the current review revealed the types and sources of evidence available on the above-mentioned question. All of the reviews would have implications for clinical training, clinical practice, the drafting of policy and regulations, and future research.

### DATA SOURCES AND SEARCH STRATEGY

The initial search was carried out in September 2020 in the following five electronic databases: Science Direct, PubMed, Scopus, Medline, and ProQuest. The databases were selected as they were deemed to be comprehensive and included publications on the use of telepractice for clinical training and SLH service delivery. The selected studies were restricted to those published in English from the year 2010 onwards, with a focus on these two specific scopes of practice: training/supervision and practice. The search consisted of the following terms: practitioner, clinician, doctor, therapist, supervision, education, telehealth, telepractice, online learning, online training, and telemedicine.

### RESOURCES

Forty-two citations were subsequently included in the analysis (see Appendix). An additional search of the aforementioned bibliographic databases was conducted in November 2020 to ensure that any additional publications post the initial search were also identified. No new publications were identified.

### CITATION MANAGEMENT

Citations were imported into the web-based bibliographic manager Endnote. A manual removal of duplicate citations was conducted using the Endnote functionality of identifying duplicates.

### ELIGIBILITY CRITERIA

The researchers adopted a two-stage screening process to evaluate the applicability of publications identified in the search. The first stage involved the inclusion of publications that contained the keywords and phrases and those broadly describing telepractice in clinical training and clinical practice. In the second stage, publications were excluded that described telepractice in areas other than healthcare; however, the reference lists from these publications were reviewed to identify additional relevant publications. Owing to limited resources for translation, only English publications were included.

### TITLE AND ABSTRACT RELEVANCE SCREENING

**Figure 1:**
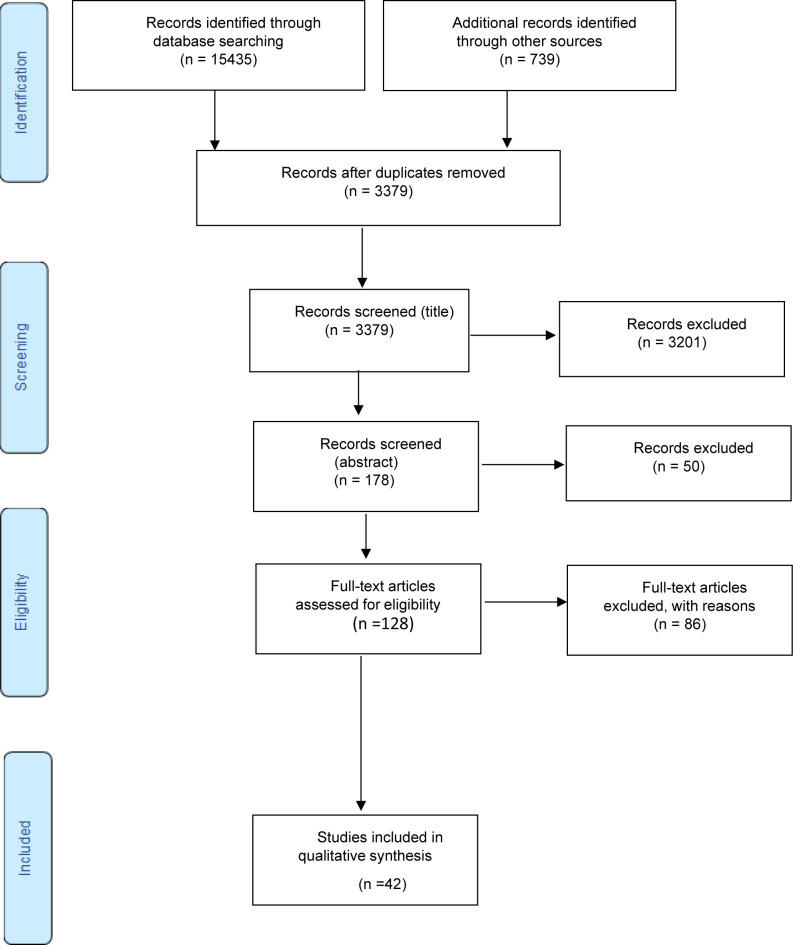
The PRISMA Flow Diagram Describing the Process of Study Selection

*Note*. Source: Moher et al. (2009)

As recommended by Arksey and O’Malley (2005): (1) the first level review examined the titles of the manuscripts; (2) the second level review examined abstracts; and (3) the third level review included entire articles (refer to Figure 1). This process eliminated articles that did not meet the study's minimum inclusion criteria. The researchers used a previously developed and pretested abstract relevance screening spreadsheet, which had been found to have high level reviewer agreement (overall kappa) greater than 0.8 (Viera & Garrett, 2005). The titles, abstracts, and entire articles were independently screened by the three researchers, with a process set up to ensure triangulation during the process of data selection and analysis. When an abstract was not available, the article underwent a full article review. The researchers communicated online to ensure that conflicts were resolved, with one author (NM) making the final decision to resolve disagreements. A high level of agreement was found with the overall kappa of 0.83. Following the data analysis, two independent reviewers (i.e., a PhD fellow and a postdoc fellow), reviewed the manuscript and the data to validate the authors’ conclusions.

### DATA CHARACTERISATION

Following the title and abstract inspection, relevant citations about telepractice in clinical training and clinical practice were extracted for later full publication reviews. The relevance of the publication was confirmed and details of the publication were recorded on a spreadsheet (i.e., author and publication year, publication title, context, clinical training versus clinical service provision, and outcomes/considerations/recommendations). The characteristics of each publication were recorded by all three researchers. Additional publications were excluded if they did not meet the minimum eligibility criteria. In adherence with Levac et al.'s (2010) framework, the researchers performed independent reviews, resolved any conflicts, and ensured consistency.

### DATA SUMMARY AND SYNTHESIS

The data were compiled in a single spreadsheet and imported into Microsoft Excel 2016 (Microsoft Corporation, Redmond, WA, USA) for descriptive narrative analysis.

A total of 16,174 studies were identified for potential analysis. In the process of collating and organising the studies, 12,795 duplicate studies were removed; thus, only 3,379 studies were considered. Of the 3,379 remaining studies, 3,251 were excluded based on the titles and/or abstracts. Consequently, 128 studies were assessed for eligibility; from these 86 were excluded as they did not meet the inclusion criteria for the current study. Finally, 42 studies were included for analysis in the current study (see Figure 1).

### ETHICAL CONSIDERATIONS

This research followed all ethical standards for studies without direct contact with human or animal subjects, including informed subjectivity and reflexivity, purposefully informed selective inclusivity, and audience-appropriate transparency (Suri, 2020).

## RESULTS AND DISCUSSION

Findings of the current review indicated an increasing use of teletraining and telepractice internationally, with very limited evidence from Africa, and/or LMICs. Studies found in these LMIC regions included those from Sri Lanka, various states across India, and Kenya (see Appendix). There was an unequal distribution between studies focusing on clinical training (26 studies) and those focusing on clinical service provision through tele-modalities (16 studies). Qualitative analysis of the studies revealed the emergence of five themes.

That limited research has been conducted on teletraining and telepractice in Africa was a significant finding; this underscored the need to accelerate the focus on these service delivery methods to increase access for both students and patients within a capacity challenged region. The heavy reliance on one-on-one, in-person direct training and/or clinical service provision was a significant drawback under normal circumstances and has been even worse during the COVID-19 pandemic.

Only one study (Carrick et al., 2017) commented on gender differences wherein women were reported to cope better with in-person learning than their male counterparts. The influence of gender on online learning is an important factor to consider as some studies have reported gender differences in online participation (Morante et al., 2017; Selwyn, 2007; Yaghmour, 2012; Yoo & Huang, 2013).

The themes that emerged from the evidence reviewed include: (1) Practice makes perfect, (2) the value of hybrid models of training and service delivery, (3) telepractice is cost-effective (4) internalization of remote clinical training and service provision, and (5) comparable modality outcomes.

### PRACTICE MAKES PERFECT

In-person treatment was perceived as effective and most preferred, pre-COVID19, followed by video service delivery. Audio only delivery was the least effective, though usable (Blumenthal et al., 2014; Martin et al., 2012). In contrast, during pandemic outbreaks, in-person learning in a physical classroom may not be possible (Mpungose, 2020).

In the current study, remote learning was reportedly less intimidating for students, health practitioners, and end-users as it allowed for greater control of the environment (Howells et al., 2019; Karaksha et al., 2013; Martin et al., 2012). These findings were confirmed by Hassenburg (2009) who asserted that, while there are benefits to being physically present and interacting with a human teacher, remote learning allows freedoms and benefits that were not previously imagined. These benefits include access for learners with disabilities and those in rural areas; cost effectiveness, convenience and flexibility; as well as ease of holding online discussions with the flexibility to pause and rewind (Hassenburg, 2009). Furthermore, remote learning allows for the training and/or clinical service provision to be performed at a pace that can be adjusted to the needs of the students and patients (Forde & Gallagher, 2020; Grewal et al., 2020; Langkamp et al., 2015; Muflih et al., 2020; Tariq et al., 2018). Moreover, in the current review telepractice services were accepted by stakeholders and students with a high satisfaction rate (Batthish et al., 2013; Cassel & Edd, 2016; Giudice et al., 2015; Langkamp et al., 2015; Likic et al., 2013; Lincoln et al., 2014; Walsh et al., 2011). Wagner et al. (2006) argued that the success of remote learning or remote interaction is dependent on the stakeholders’ satisfaction in meeting and addressing their needs and concerns. Similarly, Ramaswamy et al. (2020) reported patient satisfaction with video visits when compared to traditional in-person clinic visits.

Arguably, the process may initially be challenging, practice produces favorable results, particularly with regard to technology and administrative support, and the training of users on the ICT (Cameron et al., 2015; Likic et al., 2013). According to Meyer and Barefield (2010) administrative support is the vital foundation to a sound online education program. Similarly, the success of online learning is dependent on the skills and quality of technical support to users, without which, the ability of teachers and students to use technology will be compromised (Nawaz & Khan, 2012). The training of the site-facilitator and/or patient/caregiver is also crucial to the success of the telepractice interaction.

Concerns regarding technology were also revealed in this review (Bredfeldt et al., 2013; Langkamp et al., 2015; Lincoln et al., 2014). For instance, Lincoln et al. (2014) reported that the use of technology within the school environment increased the complexities of service delivery. However, these challenges can be mitigated by ensuring sound administrative and technology support. Goehring et al. (2012) reported that the effectiveness of remote learning and service provision is negatively impacted by limitations in technology. Therefore, the influence of technology to the success and/or failure of teletraining and telepractice cannot be overemphasized. This is consequential more so, in the initial stages, when blended learning may be used to facilitate the move to remote learning, as some end-users may not be savvy with technology.

### HYBRID TRAINING AND SERVICE DELIVERY MODELS ARE VALUABLE

The current review revealed that hybrid models (blended approaches) of clinical training, (e.g., training using e-learning to teach theoretical aspects and experiential learning for students to develop practical skills), and clinical service provision, (e.g., clinical care e-training for information counselling and therapy where physical manipulation is not required – with experiential clinical care for physical demonstration) are appreciated (Edirippulige et al., 2012).

Bredfeldt et al. (2013) conducted a study to improve providers’ effectiveness with electronic health records through blended learning, integrating concrete scenarios, hands-on exercises, and take-home materials to reinforce class concepts. The findings confirmed the value of using blended/hybrid learning as the training was well-received and for which the participants expressed a clear preference. Similarly, Guiberson et al. (2015) provided preliminary evidence on the effectiveness of a hybrid telehealth model in screening language development in children, while Chin et al. (2021) concluded that the hybrid curriculum is an innovative way to maximize learning opportunities while maintaining social distancing in the COVID-19 pandemic era.

The benefits of using hybrid models for learning have also been discussed by authors such as Hall and Villareal (2015) who documented student satisfaction with hybrid courses. This satisfaction was based on the convenience, engagement, ability to work at one's own pace, as well as comfort in expressing views. Paechter and Maier (2010) enumerated five factors associated with student satisfaction with hybrid learning: (a) clarity and structure, (b) knowledge acquisition, (c) the instructor's online expertise, (d) support from the instructor, and (e) support for cooperative learning. Esmail et al. (2009) recommend that more training in the use of technology as well as recording synchronous sessions for later review are important, as these will enhance and motivate students to engage more meaningfully. Related to motivation, the Karaksha et al. (2013) study included in the current review highlighted the need for incentivizing strategies (i.e., marketing strategies) such as emails, short message service (SMS), WhatsApp, and such, to remind and motivate learners to engage with online material and learning. When implementing online clinical training, clinical service training and marketing strategies, there should be careful consideration of the cost-effectiveness of providing such services, and interactions with funders need to occur to ensure that support is provided to patients, where needed.

### COST EFFECTIVENESS IS A “BIG WIN”

The majority of the studies highlighted cost effectiveness as one of the significant benefits of remote clinical training and service provision (Carrick et al., 2017; Chandrasinghe et al., 2020; Goehring et al., 2012; Harris & Sun, 2013; Likic et al., 2013; Thomas et al., 2021). Cost effectiveness was realized in different forms including travel time and expenses, demand versus capacity of staff and clinicians, and safe access environments. Evans and Haase (2001) discussed the benefits of remote learning; these included greater impact of money invested in training programs (i.e., value for money); significantly reduced employee travel cost and time; and the capacity to train more people (e.g., students) more often and in shorter sessions that are easier to coordinate and schedule. These benefits can easily transfer to clinical service provision and supervision.

However, one study by Moffatt and Eley (2011) concluded that telehealth is not only not cost effective, but is not a rational response as it has implications for policy, funding priorities and education and training. This is a key consideration in LMICs as resources are not easily available due to finances and poor infrastructure. To this effect, Muttiah et al. (2016) noted that LMICs have a large rural population; limited health, education and technology resources; and have a poorly performing economy. Therefore, careful attention to these factors is necessary. Sagna (2005) cautions that, although remote learning is effective in high income countries, its success in LMICs requires supplements due to poor infrastructure including limited telephone connections, poor internet bandwidth, shortage of trained personnel and limited computer skills among users.

Despite these barriers to remote learning in LMICs, current trends in Sub-Saharan Africa show that remote learning is on the rise despite persistent technological challenges. In fact, remote learning is perceived as a rational, cost-effective means to widen educational opportunities (Trines, 2018). Regarding remote service provision, Zhang and Zaman (2020) assert that telemedicine can be an efficient and cost-effective solution to address health concerns such as diagnosis and treatment of patients in remote locations. Use of a variety of information and communication technologies can minimize the difficulties and costs of traveling, save time, and provide rural populations with access to resources that are comparable to those of patients in urban areas. The current authors argue that the cost effectiveness benefits of telepractice and teletraining extends to the adoption of family-centred interventions, which have been documented to be more efficacious, especially in early intervention in SLH, wherein families can become part of the interventions without the travel costs that ordinarily prevent them from this important healthcare involvement (Maluleke et al., 2021).

### INTERNATIONALIZATION OF REMOTE CLINICAL TRAINING AND SERVICE PROVISION

Internationalizing higher education is a major goal for universities as many medical students aspire to include international experiences into their academic training (Knipper et al., 2015), and remote learning affords such opportunities. Internationalization is a significant benefit both for student training and clinical service provision. In this review, Likic et al. (2013) asserted that online teaching resources can be translated and implement internationally, and still achieve high student satisfaction rates, while decreasing administrative and cost burdens. Hansen et al. (2020) provided evidence for the feasibility of developing a multifaceted web-wide training programme for an international trial, while Cassel and Edd (2016) reported a high degree of satisfaction and improved familiarity with the use of telepractice, and an increased comfort level working with multi-cultural populations. Internationalization has benefits such as quality improvement, provision of access, competitiveness, financial profits, and the provision of a professionally relevant education that prepares all students to be interculturally proficient professionals and citizens (Wu et al., 2020). However, it may also result in unintended consequences such as ethical dilemmas. Based on the studies included in this review, it is evident that internationalization provides significant benefits both in student training and clinical service provision. However, the contextual relevance and responsiveness that are important in remote interventions (i.e., linguistic and cultural diversity issues, teaching and/or treatment, and use of multimedia options) must be carefully considered when implementing such services. Khoza-Shangase and Sebothoma (in press) argue that in South African SLH professions, internationalization can include access to the international SLH community for student clinical supervision in training platforms that are far removed from the university campuses where full-time staff are placed – and where demand-capacity challenges exist in student to staff ratios. This internationalization also has potential for the South African training programmes to provide training in the rest of Africa, where SLH training is not yet available.

### COMPARABLE MODALITY OUTCOMES

The outcomes of remote clinical teaching and clinical remote learning should mirror and match the outcomes expected from in-person training and service provision. In the current review, Berland et al. (2019) conducted a comparative analysis of online learning vs in-person training. In evaluating the educational outcomes, these authors did not find a meaningful or significant difference between in-person training and online learning. These authors concluded that online modules could provide a sustainable, convenient, and engaging approach to facilitate dissemination of lifesaving training. Similarly, Dial et al. (2019) investigated the feasibility and utility of treatment delivered via teletherapy in relation to traditional in-person treatment. At the end of the study, it was observed that there was no difference between the two approaches. Likewise, Bunker et al. (2017) and Carrick et al. (2017) observed similar outcomes between the synchronous online classroom and the traditional classroom. Various authors such as Paul and Jefferson (2019) have reported similar findings where they found no significant difference in performance between online and traditional classroom teaching with respect to modality (online vs in-person), gender, or class rank. Interestingly, Kirovska-Simjanoska (2019) concluded that students learn better through a combination of an online and traditional classroom – a hybrid model.

## CONCLUSIONS AND RECOMMENDATIONS

Clinical training and clinical service provision in the era of COVID-19 required innovative models of service delivery to ensure the health and safety of patients, clinicians, and students, as well as uninterrupted service. The use of alternative service delivery models, including teletraining and telepractice required exploring, hence the current review. This study identified 42 papers that met the predefined inclusion criteria for a scoping review. The studies that were selected were heterogeneous; therefore, attempts were not made to conduct a quantitative synthesis or meta-analysis. Nonetheless, the qualitative analysis yielded clear trends indicating the potential benefits of teletraining and telepractice under five themes.

Firstly, under “practice makes perfect,” one can conclude that training on the use of ICT as a platform is key to ensuring success and sustainability of this service delivery model. Within the African context, where task-shifting may form part of this model for clinical service provision, minimum standards of training including ICT training is important.

Secondly, under “appreciation for hybrid models of training and service delivery,” evidence suggests that carefully planned and executed training and clinical service that is blended and/or hybrid allows for flexibility and takes careful cognizance of diversity in learning style preferences as well as diversity in access and opportunities. It is important though that costs linked to this hybrid model are carefully considered and do not present as a barrier for those who do not have access to ICT infrastructure and resources, such as data.

Thirdly, as far as the “cost-effectiveness is a big win” theme is concerned, the risks-benefits evaluation of telepractice and teletraining seem to indicate more cost-effectiveness. This should be carefully considered for universal health coverage in LMICs where socio-economic challenges are significant for the population requiring access to healthcare; similarly, with those needing access to higher education.

Fourthly, under the “internationalization of remote clinical training and service provision” theme, access to clinical care as well as clinical training from the international community, as well as expanding and extending these services to the rest of Africa via e-learning and e-training is a significant benefit that needs to be explored. The analysis must bear in mind policies and regulations around internationalization in the country of origin as well as in the country of the receiver of the services. Regulations around healthcare training and delivery as well as ethical and professional codes of conduct need to be adhered to in order to ensure that professions are guided while patients are protected; as illustrated by the Health Professions Council of South Africa's mandate, for example.

Lastly, evidence under the theme “comparable modality outcomes” highlights the fact that online, teletraining, and telepractice seem to compare favourably to direct in-person delivery and/or interventions; this is a positive finding. However, the fact that better outcomes were observed in hybrid models indicates a need to explore that further, to determine factors that enhance and/or impede each model of service delivery. In SLH professions, for example, it is anticipated that equipment used for assessment and management synchronously or asynchronously (e.g., video-otoscopy, cochlear implant mapping, multi-view videofluoroscopy, etc.) would have some impact on the outcomes. These findings, as presented under the five themes, raise important implications for teletraining and telepractice globally and across health professions, but particularly in LMICs where access remains a significant challenge for both training and service provision. As evidenced by the studies included in this review, few studies were conducted in LMICs. This indicates a need for research in this area that will take careful consideration of all contextual challenges and formulate solutions that are contextually relevant and responsive.

## APPENDIX

**Table:** Studies Examined, Contexts, and Recommendations

Author(s) & Date	Publication Title	Context	Clinical		Outcomes/Considerations/Recommendations
			Training	Service Provision	
Batthish et al. (2013)	A unique, interactive and web-based pediatric rheumatology teaching module: residents’ perceptions.	Pediatric residents (n = 60) at The Hospital for Sick Children were emailed an online survey to assess prior use of online teaching modules, the utility of an online teaching module for rheumatology and which technologies should be included on such a site.	A needs assessment of core pediatric residents who will potentially be using POINTER to further understand their acceptability and requirements of this new web-based educational tool.		An interactive web-based rheumatology teaching module would be well utilized by pediatric residents. Residents showed preference for case-based teaching modules as well as multimedia modalities for learning a detailed musculoskeletal examination.
Bell & MacDougall (2013)	Adapting online learning for Canada's Northern public health workforce.	Online program which offers Internet-based continuing education modules for public health professionals.	Public Health Agency of Canada conducted 3 pilots to assess the appropriateness of the Skills Online program for Northern/Aboriginal public health workers. Module content and delivery modalities were adapted for the pilots. Adaptations included adding Inuit and Northern public health examples and using video and teleconference discussions to augment the online self-study component.		Results demonstrated that appropriate adaptations to online professional development can provide practical, accessible means for a wide range of Northern/Aboriginal public health workers to acquire core competencies for public health.
Berland et al. (2019)	Use of online opioid overdose prevention training (OOPT) for first year medical students: A comparative analysis of online vs in-person training.	First-year matriculating students in 2014 (year one), 2015 (year two) and 2016 (year three) all received OOPT. The evaluations for year two and year three were a modified version of the evaluation used in year one. The analysis includes year two (in-person group) and year three (online group).	The online training consisted of four modules covering the same five domains; “What are Opioids?” “What is Naloxone?” “Signs and Symptoms of an Opioid Overdose,” “Risk Factors for an Opioid Overdose,” and “How to Respond to an Opioid Overdose,” along with more background information on opioids and the opioid overdose epidemic. The modules were delivered using the medical school's proprietary online learning management system.		This study demonstrates that for first-year medical students, OOPT as an adjunct to BLST provided online modules obtained educational outcomes that were not meaningfully different from in-person training. With evidence that physicians are increasingly prescribing naloxone, it is even more important to train medical students to be trainers. These findings support that online modules can provide a sustainable, convenient, and engaging approach to facilitate dissemination of this important and lifesaving training.
Boet et al. (2017)	Interactive online learning for attending physicians in ultrasound-guided central venous catheter insertion.	16 attending physicians including physicians in Anaesthesiology, Critical Care, Emergency Medicine, and Internal Medicine from departments of The Ottawa Hospital who routinely perform central venous catheter insertions participated in the study on a volunteer basis.	Trained attending physicians using an online learning module as part of the continuing professional development.		Overall, the findings support the use of online CVC training to improve the CVC declarative knowledge of physicians. Deliberate practice may be necessary to complement online training to successfully reach a virtually perfect level in CVC skills for attending physicians.
Bredfeldt et al. (2013)	Training providers: Beyond the basics of electronic health records.	Two classes designed to improve providers’ effectiveness with the electronic health record. The classes focused on managing patient-level information using problem lists and medication lists, as well as efficient documentation and chart review.		Both classes used the blended learning method, integrating concrete scenarios, hands-on exercises and take-home materials to reinforce class concepts. To evaluate training effectiveness, a case–control study pre-training performance was conducted.	Ongoing information technology training is well-received by healthcare providers, who expressed a clear preference for additional training. Training improved use of two important electronic health record features that are included as part of the Meaningful Use criteria.
Brownlow et al. (2015)	Evaluation of an online training program in eating disorders for health professionals in Australia.	Expert and novice panels of health professionals inclusive of nurses, dietitians, psychologists, psychiatrists and general practitioners (GPs), in Australia participated in this study.	A pre-training questionnaire and a post training evaluation examined participants’ levels of knowledge, skill, and confidence to treat eating disorders, as well as attitudes and beliefs about people with eating disorders.		The results of this study demonstrated that the online training program was an effective tool in increasing health professionals’ level of knowledge, skill, and confidence to treat people with eating disorders. The results also demonstrated that online training reduced health professionals’ personal biases towards people with eating disorders.
Bunker et al. (2017)	The SAGES Telephone Neuropsychological Battery: Correlation with in-person measures.	50 English-speaking patients without dementia, over 70 years old and part of a cohort of patients in a prospective cohort study examining cognitive outcomes following elective surgery, were enrolled in this study.		Five well-validated neuropsychological tests were administered by telephone to each participant by a trained interviewer within 2–4 weeks of the most recent in-person interview. Tests included the Hopkins Verbal Learning Test-Revised, Digit Span, Category Fluency, Phonemic Fluency and Boston Naming Test. A General Cognitive Performance (GCP) composite score was calculated from individual subtest scores, as a Z-score.	The telephone version of a neuropsychological test battery correlated well with the in-person version, and may provide a feasible supplement in clinical and research applications.
Cameron et al. (2015)	Remote supervision of medical training via videoconference in northern Australia: A qualitative study of the perspectives of supervisors and trainees.	10 junior medical officers (Interns, Registrars) and 10 senior medical officers (Senior Medical Officers, Consultants) participated in the Townsville tele-oncology model of remote supervision via videoconference.	Supervision model for the training of junior medical officers in rural areas of North Queensland, Australia. Specifically, the perspectives of junior and senior medical officers were explored to identify recommendations for future implementation.		Remote supervision via videoconference provides readily available guidance to trainees supporting their delivery of appropriate care to patients. However, resources required for upskilling, training in the use of supervision via videoconference, administration issues and nursing support, as well as physical barriers to examinations, must be addressed to enable more efficient implementation.
Carrick et al. (2017)	Randomized controlled study of a remote flipped classroom neuro-otology curriculum.	274 (83 females and 390 males) licensed healthcare professionals in active clinical practice were randomized into two groups of 137 subjects each with careful attention to include all of the female applicants equally in each group in order to provide a greater appreciation of any gender differences in our study.	E-learning system to deliver educational materials in both live virtual classrooms as well as an on-demand system and a flipped learning classroom that complemented live learner participation in the virtual classroom.		The use of a synchronous online classroom in neuro-otology clinical training demonstrated similar outcomes to the traditional classroom. The online classroom is a low cost and effective complement to medical specialty training in Neuro-Otology. The significant difference in outcomes between males and females who attended the traditional classroom suggests that women may do better than males in this learning environment, although the effect size is moderate.
Cassel & Edd (2016)	A pedagogical note: Use of telepractice to link student clinicians to diverse populations.	Student Clinicians: Eight undergraduate university speech-language pathology students enrolled in their first clinical experience at Stockton University. Therapy recipients: Four preschool-aged children received speech and language therapy for speech sound disorders and deficits in oral language.	Student clinicians completed a 5-point ascending Likert-scale survey that targeted two parameters: (1) student-clinician familiarity and satisfaction with the use of telepractice; and (2) student-clinician familiarity and satisfaction with working with multi-cultural populations outside of their clinical experience.	Preschool-aged children received speech and language services twice weekly for eight weeks through a telepractice partnership between the university and their preschool.	Student clinician outcomes: An informal assessment of the participating student clinicians (via an ascending Likert scale) revealed a high degree of satisfaction and improved familiarity with the use of telepractice, and an increased comfort level working with multi-cultural populations. Therapy recipients’ outcomes: Based on quantitative post-evaluation findings and progress notes, all four children demonstrated improvement over the course of the academic year on their speech and language objectives. All of the children successfully transitioned to kindergarten, where any residual speech or language needs could continue to be addressed via telepractice
Chandrasinghe et al. (2020)	A novel structure for online surgical undergraduate teaching during the COVID-19 pandemic.	Online learning activity involving undergraduate medical students in Sri Lanka.	An online meeting platform with free to use basic plan and a social media platform were used in combination to communicate with the students.		Online teaching with a novel structure is feasible and effective in a resource-limited setting. Students agree that it could improve clinical interest while meeting the expected learning outcomes.
Chin et al. (2021)	Transition from a standard to a hybrid on-site and remote anatomic pathology training model during the Covid-19 pandemic.	28 clinical fellows, 35 clinical Faculty, and 22 research fellows participated in this study.	The hybrid on-site and remote anatomic pathology training model in response to the COVID-19 pandemic was implemented in the Pathology Department.		Virtual learning has become an important component of medical education, and it will become necessary for pathology departments to embrace new technologies and social media platforms. The hybrid curriculum was an innovative way to maximize learning opportunities while maintaining social distancing in the COVID-19 pandemic era. This was the first step to embrace virtual learning in postgraduate education in pathology training and warrants further exploration and development in response to changes in the landscape of pathology education.
Dial et al. (2019)	Investigating the utility of teletherapy in individuals with primary progressive aphasia.	Participants with semantic (n=10) and logopenic (n=11) PPA received lexical retrieval treatment and individuals with nonfluent/agrammatic PPA (n=10) received video-implemented script training for aphasia designed to promote speech production and fluency.	Examined the feasibility and utility of treatment delivered via teletherapy relative to treatment administered in person for individuals with PPA.		Overall, treatment outcomes were largely equivalent for individuals receiving treatment via teletherapy vs traditional, in-person delivery. Results support the application of teletherapy for administering restitutive interventions to individuals with mild-to-moderate PPA. Potential implications for using teletherapy in the treatment of cognitive-linguistic and motoric impairments in other disorders and suggestions for administering treatment via telemedicine are discussed.
Edirippulige et al. (2012)	Student perceptions of a hands-on practicum to supplement an online e-Health course.	A range of activities including introductory oral presentations, four hands-on practical exercises, observation of clinical teleconsultations, and visits to relevant sites within the hospital.	An eHealth practicum component that aimed to expose students to a range of clinically relevant learning experiences. Assessed, student perceptions of the practicum via a questionnaire.		The study showed the value of a blended learning approach, using e-learning to teach theoretical aspects and experiential learning for students to develop practical skills. Given the opportunity, students may use knowledge and skills relating to eHealth in their future practices. The emphasis on education and training of eHealth may be an important step to address the slow uptake of eHealth in the workplace. Future studies must formally assess the effectiveness of eHealth education and training.
Forde & Gallagher (2020)	Postgraduate online teaching in healthcare: An analysis of student perspectives.	12 physiotherapists, four nurses, and four other allied health scientists registered for the Online Postgraduate Certificate in Clinical Exercise.	The online Postgraduate Certificate in Clinical Exercise delivered online over one academic year via four modules with a total of 27 teaching weeks. Teaching included weekly asynchronous lectures (interactive slides with a voice over), weekly synchronous tutorials (webinars), self-directed reading, discussion board posts that were moderated by academic staff, reflective journal entries and multiple-choice questions.		This study provided evidence for the success of teaching clinical exercise online. However, workload may be perceived as heavy for students who choose to continue to work full time and there may be a need to support some online learning in practical subjects with in-person practical teaching sessions. The evidence-based recommendations provided as supplemental material to this paper may help online clinical educators and students maximize the success of their teaching and learning experiences, respectively.
Giudice et al. (2015)	Online versus in-person screening, brief intervention, and referral to treatment training in pediatrics residents.	Forty pediatric residents were randomized to receive either online or in-person training. Skills were assessed by pre- and post-training. Thirty-two residents also completed pre- and post-surveys of their substance use knowledge, attitude, and behaviours.	Pediatric residents at the University of Maryland Medical Center received ‘screening, brief intervention, and/or referral to treatment’ training as part of a Substance Abuse and Mental Health Services Administration-funded program.		Both groups demonstrated skill improvement from pre- to post assessment. Both increased their knowledge, self-reported behaviours, confidence, and readiness with no significant between group differences. Follow-up univariate analyses indicated that while both groups increased their SBIRT adherent skills, the online training group displayed more “undesirable” behaviours post training.
Goehring et al. (2012)	Evaluating the feasibility of using remote technology for cochlear implants.	Unknown		Service	Remote/distance technology for CI programming services are a viable option. To to address reimbursement limitations, evidence is needed that CI services delivered via telecommunication technology are a cost-effective alternative to in-person services. Further investigation is needed to design and validate service-delivery protocols, particularly in the areas of speech perception testing and paediatric service delivery. There are limitations in technology and optimal listening environments (i.e., sound booths) in rural locations.
Grewal et al. (2020)	Tele-health and palliative care for cancer patients: Implications for the COVID-19 pandemic.	Limited input on methodology.		Service (single case study)	Tele-palliative care offers great promise in addressing palliative and supportive care needs of patients with advanced cancer during the on-going pandemic. Continuous tele-monitoring can be used to remotely monitor crucial patient-reported outcomes such as pain and respiratory distress.
Guiberson et al. (2015)	Accuracy of telehealth-administered measures to screen language in Spanish-speaking pre-schoolers.	Spain. Eighty-two children between 37 and 69 months of age and their families participated in this study.		Service	This research provides preliminary evidence of the effectiveness of a hybrid telehealth model in screening the language development of Spanish-speaking children. A processing efficiency measure, NWR, combined with a parent survey or language sample measure can provide informative and accurate diagnostic information when screening Spanish-speaking preschool-age children for LI.
Hansen et al. (2020)	Pilot test of an online training module on near-infrared spectroscopy monitoring for the randomised clinical trial SafeBoosC-III.	100 doctors and nurses from five Neonatal Intensive Care Units across China, Spain and Denmark were invited to participate.	Training		This paper provides evidence of the feasibility of developing a multilingual web-based training program for an international trial, despite challenges such as low budget, language barriers, and possibly differences in the clinical training of staff.
Harris et al. 2020)	Using virtual site visits in the clinical evaluation of nurse practitioner students.	University of California, Los Angeles, Department of Nursing.	Virtual site visits were used to evaluate student performance and clinical observation.		Virtual site visits are feasible in most clinical settings. The lack of trained faculty greatly strains the availability for direct supervision of student training in rural areas. The benefits of virtual site visits when compared with traditional in-person clinical evaluations include decreased faculty travel time and expense.
Howells et al. (2019)	A simulation-based learning experience in augmentative and alternative communication using telepractice: Speech pathology students’ confidence and perceptions.	First year Master of Speech Pathology students at Griffith University, Australia,	First year Master of Speech Pathology students completed a 1-day simulation using a videoconferencing delivery platform with an actor portraying an adult client with motor neurone disease requiring AAC.		This study supports the use of simulation in AAC through telepractice as a means of supporting Masters level speech pathology student learning in this area of practice. Preliminary results revealed that overall, student perceptions were positive, and a majority of the students perceived the SLE, delivered via telehealth, as a valuable addition to their learning and skill development both in AAC and more broadly.
Karaksha et al. (2013)	Student engagement in pharmacology courses using online learning tools.	School of Pharmacy, Griffith University, Gold Coast campus, Australia.	Training		The provision of online teaching and learning resources were only effective in increasing student engagement after the implementation of a “marketing strategy” that included e-mail reminders and motivation.
Kolltveit et al. (2016)	Telemedicine in diabetes foot care delivery: Health care professionals’ experience.	Participants from seven home-based care services, one nurse-led primary care clinic, one medical center, and two outpatient hospital clinics attended the focus groups.		Explored health care professionals’ experience in the initial phase of introducing telemedicine technology in caring for people with diabetic foot ulcers.	Findings indicated that a telemedicine intervention enabled the participating health care professionals to approach their patients with diabetic foot ulcer with more knowledge, better wound assessment skills, and heightened confidence. Furthermore, it streamlined the communication between health care sites and helped treat patients in a more holistic way.
Kuziemsky et al. (2020)	Ethics in telehealth: Comparison between guidelines and practice-based experience – the case for learning health systems.	IMIA Telehealth Working Group (WG) was made of more than 60 members from across the globe working in the telehealth arena as entrepreneurs, promoters, academics, and practitioners. Cultural and regional differences were discussed in relation to Sri Lanka, United States, Columbia, and Argentina.		Service/ethics	Teletherapy results in changes in patient-provider communication patterns, access to care delivery services, and how patients interact with telehealth tools. Unintended consequences may occur from telehealth usage. A strategy going forward must be to embrace the notion of learning health systems (LHSs) to better understand the new interactions and relationships that develop from telehealth usage in different contexts. There was a difference in priorities expressed by practitioners and the content of ethical guidelines.
Langkamp et al. (2015)	Telemedicine for children with developmental disabilities: A more effective clinical process than office-based care.	4 parent/child dyads from Akron Children's Hospital in Ohio, USA.		Service	Most parents expressed a high level of satisfaction with the program. Benefits identified included decreased stress to the child and the parents and an increased likelihood of a successful medical examination due to greater cooperation by the child. Visits using asynchronous or “store and forward” telemedicine technology may be superior in some situations by allowing the visit to be performed at a pace that can be adjusted to the needs of the child with developmental disabilities (DD). The authors suggest that more research in the use of asynchronous telemedicine for children and youth with DD, is needed, particularly for children with DD and challenging behaviors.
Likic et al. (2013)	Online learning applied to a course on rational therapeutics: An international comparison between final year students of two medical schools.	North America (University of Michigan) and Croatia (University of Zagreb).	In 2009, an electronic problem-based therapeutics course developed at the University of Michigan Medical School (UMMS) was translated and adapted for use at the University of Zagreb Medical School (UZMS).		It is possible to adapt and translate online teaching resources and implement them internationally in different countries and health care systems, achieving similar high student satisfaction rates while decreasing administrative and cost burdens. Web based learning may have great potential to offer a cost effective and safe environment in which prescribing skills can be improved.
Lincoln et al. (2015)	Multiple stakeholders’ perspectives on teletherapy delivery on speech pathology services in rural schools: A preliminary qualitative investigation.	New South Wales, Australia.		Service	Nine children received speech pathology sessions delivered via Adobe Connect® web-conferencing software. Despite frequent reports of difficulties with technology, teletherapy delivery of speech pathology services in schools was highly acceptable to stakeholders. However, the use of technology within a school environment increased the complexities of service delivery. Stakeholders suggested that implementation of teletherapy within rural school settings must incorporate processes that support the development of partnerships at a distance.
Martin et al. (2012)	An evaluation of remote communication versus face-to-face in clinical dental education.	United Kingdom, University of Sheffield, Department of Dentistry.	Each communication modality was tested in exercises in a random order between an expert and a student, from a cohort of 15 senior clinical Dental under-graduate students.		Physical in-person learning was perceived to be a more effective communication modality for clinical case-based discussions between a learner and an expert. However, remote, internet-based discussions enabled a more relaxed discussion environment. Good eye contact was observed in the three modalities, but less so in-person.
Moffatt & Eley (2011)	Barriers to the up-take of telemedicine in Australia - a view from providers	10 established expert providers of telemedicine services, combining Australian and international experience. Their roles/occupations included: moderators, academics, medical specialists, internet technology specialists, educators and program developers.		Provided an update on barriers to the uptake of telemedicine, in Australia, by the providers of telemedicine services.	These results raise issues in the domains of policy, funding priorities, and education and training. This indicates an inter-related set of challenges that would require a targeted multifaceted approach to address. The results suggest that not using telemedicine is, in the current climate, a rational response - it is quicker, easier and more cost-effective not to use telemedicine.
Mohan et al. (2017)	A survey of telepractice in speech-language pathology and audiology in India.	A total of 205 speech-language pathologists and audiologists completed the survey, from different states across India. A questionnaire was developed to gather responses from two groups. Group I consisted of speech-language pathologists and audiologists engaged in telepractice. Group II included speech-language pathologists and audiologists not engaged in telepractice.	Twenty closed ended questions were included to study the perspectives of speech-language pathologists and audiologists on telepractice. Questions addressed four content areas: perceptions of professionals about service delivery through telepractice; characteristics of telepractice service; training and research in telepractice; and policies and guidelines for telepractice.		The findings of the survey endorse the feasibility of providing speech-language and audiology services through telepractice. The results reflect the opinion of the professionals for whom telepractice is a plausible mode of service delivery. The extensive technology available to healthcare providers such as speech-language pathologists and audiologists, and their clients, should be used to its fullest extent to enable better quality care in settings and regions where the physical presence of expertise is unavailable.
Muflih et al. (2020)	Online education for undergraduate health professional education during the COVID-19 pandemic: Attitudes, barriers, and ethical issues.	A total of 1,210 participants agreed to complete the online survey questionnaire.	Explored whether the pandemic of COVID-19, which requires universities to rapidly offer online learning, will affect attitudes about online education for undergraduate health sciences students. Also, it investigated the barriers for using online tools.		Although the pandemic of COVID-19 appeared as uncommon catalyst for promoting eLearning, further research is needed to assess whether learners are ready and willing to make greater use of online education to obtain high quality teaching and learning opportunities; this could alter educators’ and students’ attitudes and impression, and subsequently the general themes of online education.
Mukhtar et al. (2020)	Advantages, limitations and recommendations for online learning during COVID-19 pandemic era.	12 faculty members and 12 students from University College of Medicine and University College of Dentistry, Lahore participated. Four focus group interviews, two each with the faculty and students of medicine and dentistry were carried out.	Explored faculty and students’ perceptions about online learning modalities, its advantages, limitations, and recommendations.		The study supports the use of online learning in medical and dental institutes, and considers its various advantages. Online learning modalities encourage student-centred learning and they are easily manageable during this lockdown situation.
Pimmer et al. (2013)	Smartphones as multimodal communication devices to facilitate clinical knowledge processes: Randomized controlled trial.	42 medical students in a master's program.	Determined the effects of different synchronous smartphone-based modes of communication, such as (1) speech only, (2) speech and images, and (3) speech, images, and image annotation (guided noticing) on the recall and transfer of visually and verbally represented medical knowledge.		The results show (1) the value of annotation functions for digital and mobile technology for inter-clinician communication and medical informatics, and (2) the use of guided noticing (the integration of speech, images, and image annotation) leads to significantly improved knowledge gains for visually represented knowledge. This is particularly valuable in situations involving complex visual subject matters, typical in clinical practice.
Rafferty et al. (2019)	Pilot testing of an online training module about screening for acute HIV infection in adult patients seeking urgent healthcare.	Two pilot test sessions were undertaken. The first included 17 clinical officers (COs) working at the outpatient department of Malindi District Hospital, a coastal sub-county hospital. The second session was organized at a CO training school in Mtwapa and delivered to 28 final year students.	Assessed knowledge gained and areas for improvement of the module and to assess potential for online provision of both the training module and training COs in the use of a screening algorithm to identify young, at-risk adults who should be tested for acute and early HIV.		Acute HIV infection (AHI) is a common and important condition of which clinical officers in Coastal Kenya have poor knowledge. A self-directed learning module was successful at improving knowledge about AHI; however, the cohort struggled to utilize the screening algorithm. The pilot-tested module has been revised and adapted for online delivery, and further evaluations and refinement are planned. Clinical knowledge of AHI is key to improving diagnosis.
Schlenz et al. (2020)	Students’ and lecturers’ perspective on the implementation of online learning in dental education due to SARS-CoV-2 (COVID-19): a cross-sectional study.	A total of 242 (166 female, 69 male) students completed the questionnaire.	Evaluated the students’ and lecturer's perspective toward the new online learning courses through two online questionnaires (one for students and one for lecturers) in the challenging time of the SARS-CoV-2 pandemic.		Within the limitation of this study, students and lecturers showed a predominantly positive perspective on the implementation of online learning, providing the chance to use online learning even beyond COVID-19 in the future curriculum.
Steventon et al. (2016)	Effect of telehealth on hospital utilisation and mortality in routine clinical practice: A matched control cohort study in an early adopter site.	716 telehealth patients were recruited from the community, general practice and specialist acute care, between June 2010 and March 2013. Patients had chronic obstructive pulmonary disease, congestive heart failure or diabetes, and a history of associated inpatient admission.		Observational study of a mainstream telehealth service, using person-level administrative data.	If telehealth is pursued, it may be desirable to create information systems to enable these services to respond to learning and to seek to improve their effectiveness over time. The methods used in this study, including linkage to administrative data, selection of matched control groups and sensitivity analysis, could be adapted to enable effectiveness to be tracked in close to real time.
Tariq et al. (2018)	Mobile detection of autism through machine learning on home video: A development and prospective validation study.	A mobile web portal for video raters to assess 30 behavioural features (e.g., eye contact, social smile) that are used by eight independent machine learning models to for identifying ASD. Each of these had >94% accuracy in cross-validation testing and subsequent independent validation from previous work.		Analysed item-level records from 2 standard diagnostic instruments to construct machine learning classifiers optimized for sparsity, interpretability, and accuracy. The present study prospectively tested whether the features from these optimized models can be extracted by blinded non-expert raters from 3-minute home videos of children with and without ASD to arrive at a rapid and accurate machine learning autism classification.	These results support the hypothesis that feature tagging of home videos for machine learning classification of autism can yield accurate outcomes in short time frames, using mobile devices. Further work will be needed to confirm that this approach can accelerate autism diagnosis at scale.
Thomas et al. (2021)	Synchronous telemedicine in allergy: Lessons learned and transformation of care during the COVID-19 pandemic.	A total of 537 synchronous telephone encounters were conducted.		Described the outcomes of the use of synchronous telemedicine for outpatient consultations in a tertiary adult allergy center.	Telemedicine can transform the current models of allergy care. Screening criteria for selecting suitable new patients are required. A telemedicine-based drug allergy service model can be more time- and cost-effective and improve patient access to specialist care.
Walsh et al. (2011)	Online teaching tool simplifies faculty use of multimedia and improves student interest and knowledge in science.	Sixty-three students registered to use the Online Multimedia Teaching Tool (OMTT).	Employed Adobe ColdFusion– and Adobe Flash–based system for simplifying the construction, use, and delivery of electronic educational materials in science. The OMTT in Neuroscience was constructed from a ColdFusion based online interface, which reduced the need for programming skills and the time for curriculum development. The OMTT in Neuroscience was used by faculty to enhance their lectures in existing curricula.		The OMTT was rapidly adapted by multiple professors, and its use by undergraduate students was consistent with the interpretation that the OMTT improved performance on exams and increased interest in the field of neuroscience
Ye et al. (2016)	A telemedicine wound care model using 4G with smart phones or smart glasses: A pilot study.	29 males and 1 female presented with varicose veins and wounds in leg (n=16), ankle (n=8) and foot (n=6).		A new telemedicine wound care model was set-up using 4th-generation mobile communication technology. Standards (4G) net with smart glasses or smart phones between Shanghai and Jinhua, which are 328km apart, to conduct a cohort study.	In general, this wound care model is an interactive, real-time, and remote care strategy designed to improve health care service. It is an innovative process to implement telemedicine, which can be carried out to change wound dressings or do operations with the help of expert opinions. Both local surgeons and patients showed good acceptance of this model.
